# The role of difficulties in emotion regulation on non-suicidal self-injury and suicide attempts: a cross-sectional study of Chinese adolescents

**DOI:** 10.1038/s41598-025-91962-5

**Published:** 2025-07-01

**Authors:** Hao Xu, Dianying Liu, Xuejing Xu, Jinghan Wang, Bixin Wang, Wenkai Zheng, Yan Chen, Wei Qu, Yunlong Tan, Zhiren Wang, Yanli Zhao, Shuping Tan

**Affiliations:** 1 Huilongguan Clinical Medical School Beijing Huilongguan Hospital, Peking University, Beijing, 100096 China; 2 Ganzhou City Key Laboratory of Mental Health, The Third People’s Hospital of Ganzhou City, Ganzhou, 341000 Jiangxi China; 3 Temple University, Philadelphia, PA 19122 USA; 4 Inner Mongolia Medical University, Hohhot, 010110 Inner Mongolia China

**Keywords:** Adolescent, Non-suicidal self-injury, Suicide attempts, Difficulties in emotion regulation, Depression, Psychology, Psychiatric disorders

## Abstract

Non-suicidal self-injury (NSSI) and suicide attempts (SA) among adolescents are issues of widespread concern. Non-suicidal self-injury and attempted suicide are both risk factors for suicide, and difficulties in emotion regulation (DER) may play a crucial role in their occurrence. Therefore, our study aims to investigate the role of DER in NSSI and SA among adolescents, which is of substantial clinical importance for the prevention and intervention of adolescent suicidal behaviors. A web-based questionnaire survey was administered to 9140 Chinese adolescents aged 12–18 years. Utilizing chain mediation and a threshold effect model, we investigated the impact of DER in relation to NSSI and SA among adolescents. DER had a direct and indirect effect on NSSI severity by amplifying depressive symptoms, which consequently increased the severity of NSSI. Furthermore, DER directly elevated the risk of SA by exacerbating depressive symptoms and NSSI severity. We observed a threshold effect, whereby the DER significantly increased the severity of NSSI after reaching a threshold score of 87. Our study provides valuable insights into the psychological mechanism of DER in relation to NSSI and SA among Chinese adolescents, underscoring the importance of intervention and prevention strategies aimed at addressing these underlying difficulties. These findings contribute to research on adolescent suicide and fill a knowledge gap in this field.

**Supplementary Information:**

The online version contains supplementary material available at 10.1038/s41598-025-91962-5.

## Introduction

Adolescent suicide is the third leading cause of death among 10–19 years old worldwide and is on the rise^1^. Suicide attempts (SA) are an important factor in death by suicide^2^. Non suicidal self-injury (NSSI) is significantly associated with SA in adolescents^3,4^. The global incidence of NSSI in adolescents is approximately 19.5%, and for Chinese adolescents (13–18 years of age), it is approximately 27.4% and occurs at an earlier age^5,6^. Both NSSI and SA are risk factors for suicidal behaviors, which often occur simultaneously and have common influencing factors^7,8^. Current evidence suggests that adolescent NSSI behavior serves to alleviate negative emotions^9^. The cognitive emotional model (CEM)^10^, four-function model^11^, emotional cascade model (ECM)^12^, experiential avoidance model (EAM)^13^, and benefits and barriers model^14^ all highlight the importance of emotion regulation. The ECM suggests that difficulties in emotion regulation (DER) may lead to the accumulation of negative emotions, which in turn may trigger or exacerbate NSSI and SA behaviors. This theory argues that adolescents’ DER not only directly affects how they cope with emotions but also potentially worsens their negative emotions and suicide risk by triggering emotional cascades^12^. The EAM further emphasizes that adolescents’ strategies of avoiding painful emotions during emotion regulation may intensify emotional distress, thereby increasing the occurrence of NSSI and SA^13^. The CEM suggests that adolescent NSSI may be the result of internal emotional conflicts and cognitive distress. NSSI, as a form of emotion regulation, can temporarily alleviate emotional pressure and may also worsen psychological issues^10^.

DER is a prominent feature in adolescents’ mental health, influencing their NSSI and SA^15^. Adolescents’ DER is closely related to NSSI and makes it difficult for adolescents to effectively deal with negative emotions, resulting in increased and prolonged negative emotions, for which NSSI is adopted as a strategy for emotional regulation^16^. DER in adolescents is also an important predictor of SA, affecting NSSI and SA in adolescents through many factors^17^. DER may increase the risk of NSSI and SA in adolescents via the occurrence or aggravation of depression^18^. Adolescence is also a period with a high incidence of emotional problems such as anxiety and depression^19^. Patients with depression who engage in NSSI behavior often have a higher risk of suicide^20^.

During a 1-year follow-up period, 10.9% of adolescents with NSSI behavior had at least one instance of SA^21^. DER can also increase the risk of mobile phone addiction among adolescents^22^. Mobile phone use can provide temporary relief and escape but makes teenagers more likely to experience negative emotions, thus increasing their risk of developing NSSI and SA behaviors. DER also reduces adolescents’ empathy in interpersonal communication, leading to a reduction in effective social support, which further aggravates DER^23^. Interventions for DER can alleviate the degree of NSSI in adolescents and are one of the ways to prevent adolescent suicide^24^.

In recent years, more attention has been paid to the mental health of Chinese adolescents. The “Healthy China Action (2019–2030)” issued by the National Health Commission of China in July 2019 proposed to “carry out mental health promotion actions and primary and secondary school mental health promotion actions,” which set phased goals for 2022 and 2030 for adolescent mental health^25^. As mental health problems increase among Chinese adolescents, more attention has been paid to NSSI and SA^26–28^. However, the mechanisms by which DER affects NSSI and SA among adolescents have not received sufficient attention. Therefore, this study aims to verify the role of DER in NSSI and SA in a sample of Chinese adolescents, with a view to enriching research in this field. We hypothesize that DER may increase the risk of SA and the severity of NSSI. Additionally, DER may increase the risk of SA by depression, NSSI, mobile phone addiction, and empathy. Furthermore, DER may have a significant threshold effect on the risk of SA in adolescents with NSSI and on the severity of NSSI in adolescents with NSSI.

## Methods

### Participants

A questionnaire survey was distributed to 9140 participants aged 12–18 years from three high schools in Jiangxi Province, China, from September to December 2022. Informed consent was obtained online from both participants and their guardians. Students who opted out of participation were automatically removed from the survey, and their information was not retained. The participants responded to questionnaires on their mobile phones. The survey included questions on demographics and mental health. This study was approved by the Ethics Committee of Beijing Huilongguan Hospital (2021-24-Ke). We confirm that all methods were performed in accordance with the relevant guidelines and regulations.

### Assessments

#### Non-suicidal self-injury

We used the Adolescent Non-Suicidal Self-Injury Assessment Questionnaire to assess incidence of NSSI among the participants^29^. The questionnaire comprised 12 questions rated on a 5-point Likert scale, with each item corresponding to a specific self-injuring behavior. The severity of NSSI was correlated with the total score. This questionnaire has been proven to be effective for Chinese adolescents^29^. The Cronbach’s α coefficient was 0.940 in this study.

#### Suicide attempts

Participants were invited to answer two questions^30^, including “Have you ever made a suicide attempt in your lifetime?” and “Have you made any suicide attempts in the past 12 months?” If the response to either of these items was affirmative, the individual was categorized as having a history of suicide attempts.

#### Difficulties in emotion regulation

We used the Chinese version of the difficulties in emotion regulation scale to determine the adolescents’ degree of difficulty regulating their emotions^31^. The 36-items were rated on a 5-point Likert scale. The higher the total score, the more difficult it was to regulate emotions. This questionnaire has been proven to be useful for Chinese adolescents^30^. The Cronbach’s *α* coefficient was 0.962 in this study.

#### Depression

We used the Patient Health Questionnaire-9 (PHQ-9) to assessed participants’ depressive state. This questionnaire has been proven to be effective for Chinese adolescents^32,33^. Depression severity increased as the score increased. The questionnaire contained nine items rated on a 4-point Likert scale. The Cronbach’s *α* coefficient was 0.882 in this study.

#### Mobile phone addiction

We used the Chinese version of the Mobile Phone Addiction Tendency Scale (MPATS) to assess participants’ level of mobile phone addiction^34^. This questionnaire has been proven to be useful for Chinese adolescents^30^. A higher total score indicates greater severity of mobile phone addiction. The questionnaire contained 16 items rated on a 5-point Likert scale. The Cronbach’s *α* coefficient was 0.938 in this study.

#### Empathic capacity

We used the Chinese Interpersonal Reactivity Index (C-IRI) to assess participants’ empathic capacity^35^. This questionnaire has been proven to be useful for Chinese adolescents^30^. The scale consists of 28 items measured on a 5-point Likert scale, where higher total scores indicate greater empathic ability. The Cronbach’s *α* coefficient was 0.934 in this study.

### Statistical analysis

The initial sample size was 9140. To eliminate the interference of extreme or abnormal values and ensure data accuracy and reliability, we excluded samples with response times exceeding three standard deviations from the mean, resulting in 7030 remaining samples. Participants with response times deviating by more than three standard deviations from the mean might have been interrupted or overly decisive during the answering process, affecting data reliability. Subsequently, we removed any rows with one or more missing values in the answers, leaving 6959 samples. The NSSI scale was divided into yes or no answers, with a threshold score of > 1 for “yes” and a total score of 0 for “no.” In the final statistical analysis, 2496 samples with a history of NSSI were included. Since the variables did not have a normal distribution, we analyzed continuous variables using the Mann–Whitney U test and categorical variables using the chi-square test to compare differences between groups. Owing to the methodological limitations of our study, we used Harman’s single factor test to measure common method bias.

We conducted Spearman correlation analysis to assess the associations between the DERS, PHQ-9, MPATS, IRI, NSSI, and SA. Additionally, stepped-linear regression was used to identify correlations between DERS, PHQ-9, MPATS, IRI, and NSSI. We adopted a stepped-logistic regression model for analysis associations between DERS, PHQ-9, MPATS, IRI, NSSI, and SA.

In a chain mediation analysis, DERS was the independent variable, the PHQ-9 and NSSI mediating variables, and SA the dependent variable. The analysis also controlled for the effects of MPATS, IRI, and sex as covariates. We employed maximum likelihood estimation and conducted 5000 bootstrap resampling iterations to ensure the robustness of the results. Standardized coefficients and confidence intervals obtained using the bootstrap method were used to assess the statistical significance and relative importance of relationships among variables in the model. The 95% confidence interval did not include zero and was considered statistically significant^36^. As the dependent variable was a binary variable, we used standardized coefficients^37^.

Nonlinear associations were fitted and presented as smoothing splines based on generalized additive models^38^ between NSSI as the dependent variable and DERS as the independent variable, while PHQ-9, MPATS, IRI, and sex were controlled for. We utilized a recursive algorithm to compute the inflection point, followed by threshold effect analysis using a segmented regression model. Additionally, we performed a log-likelihood ratio test to compare the one-line linear regression model with the two-piecewise linear model. A 2-tailed *P* value of < 0.05 was considered statistically significant. We also performed a threshold effect analysis using SA as the dependent variable. Statistical analyses were performed using IBM SPSS Statistics 27, Mplus 8.3, and R 4.3.0.

## Results

### Characteristics of participants

Table 1 shows that the median age of the 6959 participants was 16 years, with 53.89% females and 10.80% reporting a history of suicide attempts. Further, 2496 adolescents (35.87% of the sample) reported engaging in non-suicidal self-injury at least once. Harman’s single factor analysis revealed that the variance of the first common factor was 36.15%, which was below 40%, indicating the absence of serious common method bias (Supplementary Table S1). Spearman correlation analysis showed significant correlations between DERS, PHQ-9, IRI, MPATS, NSSI, and SA (Supplementary Table S2).

**Table 1:** Characteristics of participants.

	No SA N = 6209 (89.2%)	SA N = 750 (10.8%)	*χ*^2^/Z	*p*
	N (%)/Median (Q1, Q3)	N (%) median (Q1, Q3)		
Sex (female)^a^	3224 (51.92%)	526 (70.13%)	89.29	< 0.001
Age^b^	16 (15, 16)	16 (15, 16)	− 2.45	0.01
NSSI behavior (binary)^a^	1921 (30.94%)	575 (76.67%)	608.30	< 0.001
PHQ-9 total^b^	5 (2, 9)	11 (7, 16)	− 23.69	< 0.001
IRI total^b^	86 (78, 94)	94 (87, 101.25)	− 15.80	< 0.001
MPATS total^b^	38 (29, 47)	48 (39, 57)	− 17.71	< 0.001
DERS total^b^	83 (75, 92)	100 (88, 114)	− 22.41	< 0.001
NSSI Behavior total^b^	0 (0, 1)	5 (1, 13)	− 29.11	< 0.001

*NSSI* non-suicidal self-injury, *SA* suicide attempts, *No SA* no suicide attempts.

^a^Chi-square test.

^b^Mann–Whitney U test.

The results of the stepwise linear regression with NSSI as the dependent variable indicate a significant positive correlation between DERS, PHQ-9, and NSSI, while MPATS, IRI, and sex were excluded. The results of the stepwise logistic regression model with SA as the dependent variable suggest that DERS, PHQ-9, and NSSI significantly increased the risk of SA, with IRI and MPATS being excluded. Additional details are provided in Supplementary Tables S3 and S4.

### Association between DERS and SA: a chain mediation test

**Fig. 1:**
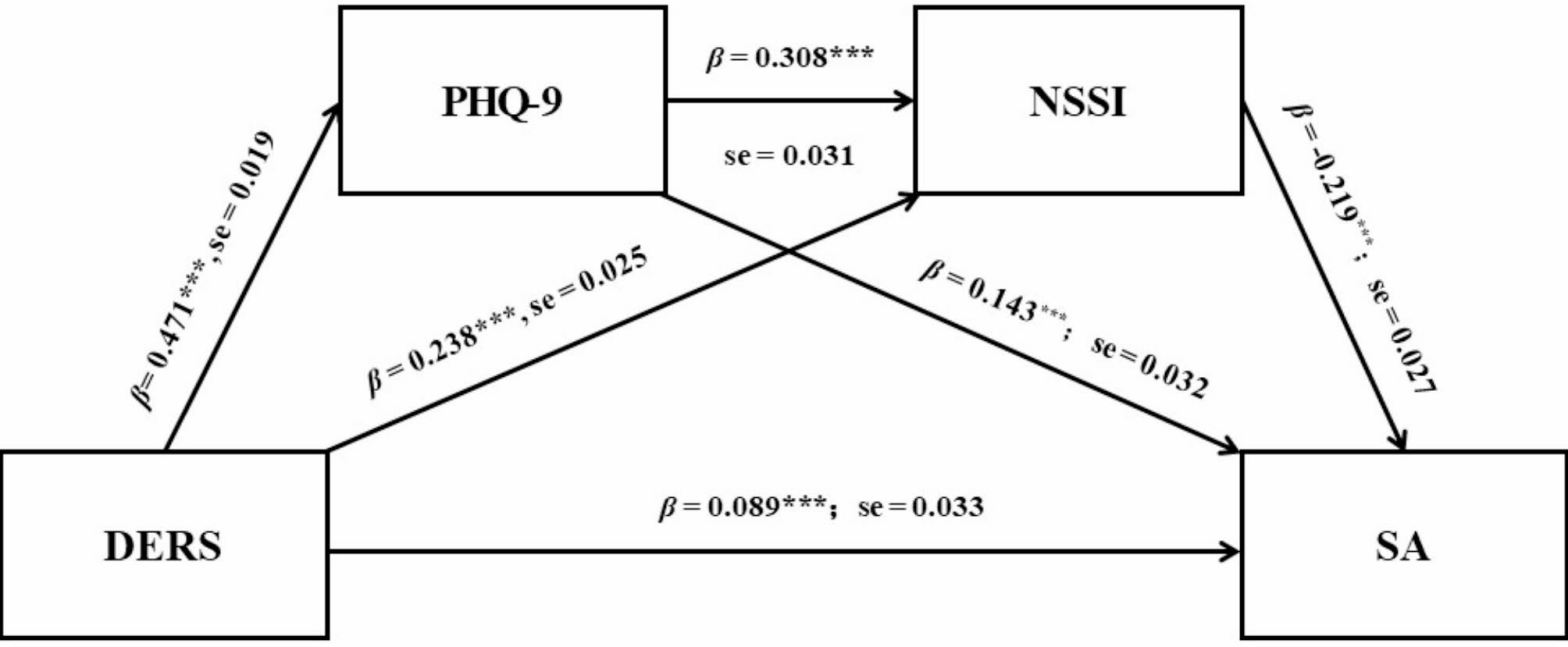
The chain-mediated model of the effect of DERS on the risk of NSSI and SA. *DERS* difficulties in emotion regulation scale, *SA* suicide attempts, *PHQ-9* patient health questionnaire-9, *NSSI* non-suicidal self-injury assessment questionnaire; ***p < 0.001; *β*, Standardization coefficient.

Table 2 shows the chain mediation analysis, which demonstrates that the direct effect size is significant, with an effect size of 0.089. Furthermore, the mediating effect of PHQ-9 on the relationship between DERS and SA is significant, with an effect size of 0.067 at the 95% confidence interval. Similarly, the mediating effect of NSSI on the relationship between DERS and SA is also significant, with an effect size of 0.056 at the 95% confidence interval. Additionally, the combined effect of PHQ-9 and NSSI on the relationship between DERS and SA is significant, with an effect size of 0.032. The total effect is 0.243, with a total indirect effect of 0.155. These results indicate that DERS can directly influence SA and also indirectly affect SA through PHQ-9 and NSSI, as illustrated in Fig. 1.

**Table 2:** Chain-mediated model of DERS on the risk of NSSI and SA.

Effect		*β*	*SE*	Bootstrap 95% CI
Direct	DERS → SA	0.089	0.033	[0.025, 0.156]
Specific indirect	DERS → PHQ-9 → SA	0.067	0.015	[0.038, 0.098]
	DERS → NSSI → SA	0.056	0.009	[0.040, 0.075]
	DERS → PHQ-9 → NSSI → SA	0.032	0.005	[0.022, 0.044]
Total indirect		0.155	0.018	[0.121, 0.374]
Total		0.243	0.029	[0.186, 0.301]

*DERS* difficulties in emotion regulation scale, *SA* suicide attempts, *PHQ-9* patient health questionnaire-9, *NSSI* non-suicidal self-injury assessment questionnaire, *β* standardization coefficient.

### Smoothing splines and threshold effect analysis

#### Threshold effect of NSSI severity

**Fig. 2:**
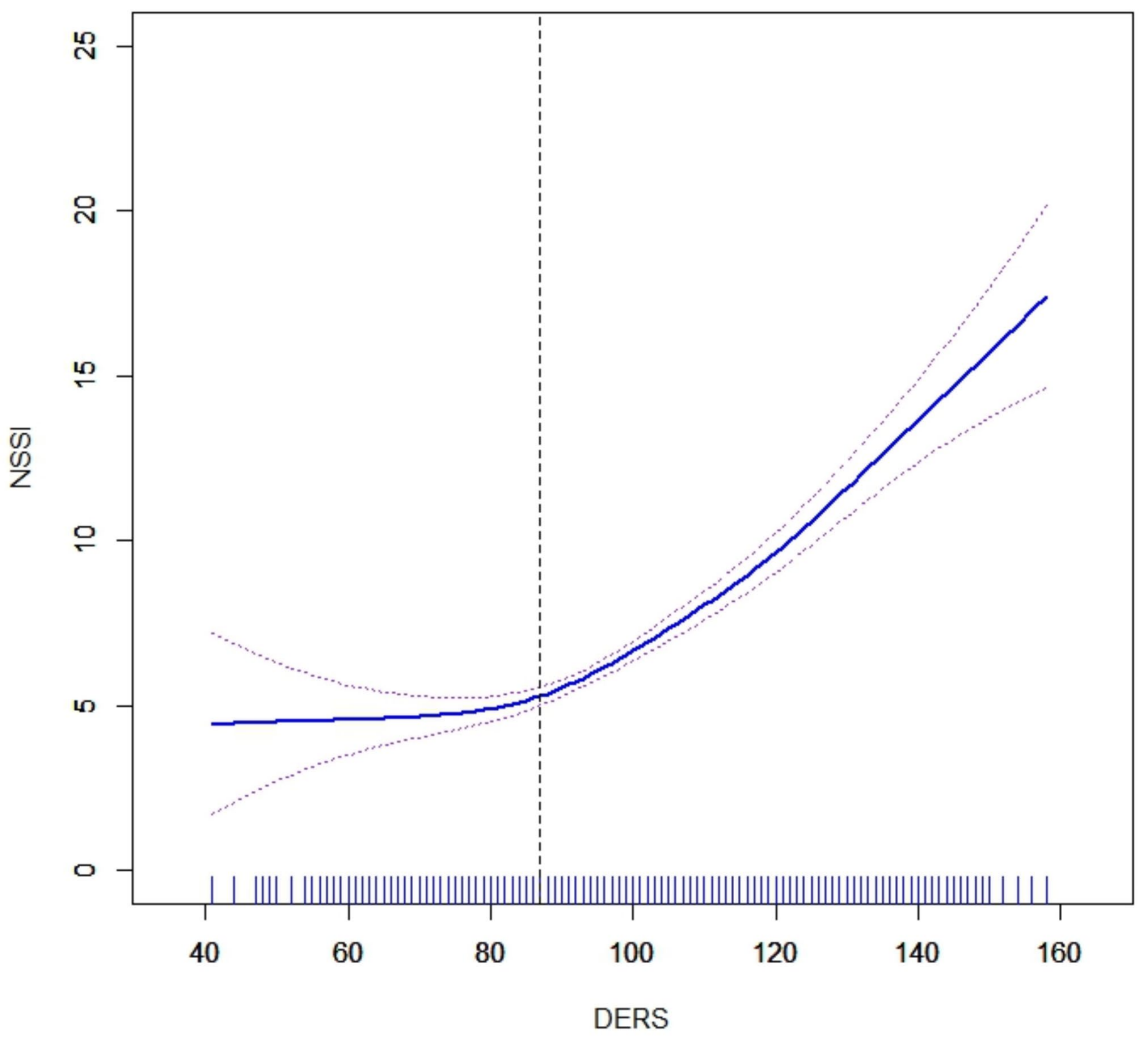
The smoothing splines of NSSI with DERS. *DERS* difficulties in emotion regulation scale, *NSSI* non-suicidal self-injury assessment questionnaire.

Figure 2 represents the DERS score; the ordinate represents the NSSI score; the solid blue line is the fitted smooth curve, and the dashed purple line represents the 95% confidence interval. The nonlinear relationship between DERS and NSSI severity shows that NSSI severity increases with an increase in DERS scores, rising slowly at first before increasing rapidly, indicating a nonlinear relationship. Table 3 presents the threshold effect results analyzed using a segmented regression model with a breakpoint at 87 points. Before 87 points, the influence of DERS on NSSI is not significant (*β* = − 0.006, *p* = 0.090, *p* > 0.05). However, after 87 points, DERS significantly affects NSSI (*β* = 0.353, *p* < 0.001); this indicates that for every one-unit increase in DERS, NSSI increases by 0.353. The difference between the two models is significant (*β* = 0.359, *p* < 0.001), with a log-likelihood ratio test yielding *p* < 0.001, which suggests that the segmented linear regression model outperforms a single linear regression model. As shown in Supplementary Table S5, the participants were grouped based on the breakpoint. The scores for the 12 types of self-harm behaviors were subjected to Mann–Whitney U tests, and the results revealed that the top three differences in scores were for “scratching,” “pinching,” and “hitting,” followed by cutting, stabbing, and hitting hard objects.

**Table 3:** Analysis of threshold effect on the NSSI.

Models	β	95% CI	*p*
Model 1			
Single linear regression coefficient	0.254	(0.210, 0.299)	< 0.001
Model 2			
Turning point (K)	87		
< K (a)	− 0.006	(− 0.108, 0.094)	0.090
≥ K (b)	0.353	(0.297, 0.409)	< 0.001
Difference in effects between coefficients b and a	0.359	(0.234, 0.485)	< 0.001
Log-likelihood ratio test			< 0.001

A 2-tailed *P* value of log-likelihood ratio test.

### Threshold effect analysis with risk of SA

Using a two-part logistic regression model, with SA as the dependent variable, the analysis suggests that the risk of SA increases with higher DERS scores. The log-likelihood ratio test yielded *p* = 0.082, which indicates that the segmented model lacks statistical significance (*p* > 0.05). Additional details are presented in Supplementary Fig. S1 and Table S6.

## Discussion

This study investigated the impact of DER on NSSI and SA in Chinese adolescents. Of the 6,959 participants surveyed, 10.80% reported a history of suicide attempts, and 35.87% had engaged in at least one episode of NSSI. Of these participants, 575 (8.26% of the total sample) reported both NSSI and a history of SA. Among participants who engaged in NSSI, 23.03% had a history of SA, and the proportion of females was higher than that of males, which is consistent with the findings of Wang et al.^39^ The prevalence of NSSI and SA in adolescents deserves further attention.

The findings indicated that adolescent NSSI and SA were influenced by many factors^40^, and Spearman correlation analysis confirmed significant correlations between DERS, PHQ-9, IRI, MPATS, NSSI, and SA. Chain mediation analysis revealed that in the pathway DERS → PHQ-9 → SA, DERS is positively associated with PHQ-9; this indicates that DER can exacerbate the severity of depression, thereby increasing the risk of SA. In the pathway DERS → NSSI → SA, DERS is positively associated with NSSI; this suggests that DER can increase the severity of self-injury, thereby raising the risk of SA. In the pathway DERS → PHQ-9 → NSSI → SA, DERS is positively associated with both PHQ-9 and NSSI; this indicates that DERS can exacerbate the severity of depression, which in turn worsens the severity of NSSI, thus increasing the risk of SA. In the pathway DERS → SA, DERS is positively associated with SA; this demonstrates that DERS can directly increase the risk of SA. Therefore, adolescent DER not only directly exacerbates the severity of NSSI^17^, but it also indirectly increases the severity of NSSI by worsening depression. It directly increases the risk of SA among adolescents and intensifies both depression and NSSI, thereby indirectly elevating the risk of SA^41^.

Threshold effect analysis revealed a threshold effect of adolescent DER on the severity of NSSI, with a turning point at 87 points. Beyond 87 points, the severity of NSSI increased significantly. This indicates that in clinical practice, more attention should be given to adolescents with a history of NSSI and a score of 87 points or above on the DER scale to prevent exacerbation of NSSI and lower the risk of future SA. Furthermore, when comparing the NSSI behavior of adolescents before and after the 87-point threshold, we found that “scratching,” “pinching,” and “hitting” were the top three behaviors with the most significant differences. These behaviors should receive more attention to prevent the escalation of NSSI severity and consequent increase in the risk of SA among adolescents. However, we found no threshold effect of DER on the risk of SA among adolescents with NSSI. Nonetheless, we observed a significant linear relationship, which indicates that among adolescents with a history of NSSI, the risk of SA increases linearly with the level of DER. Therefore, interventions targeting DER in adolescents who have engaged in NSSI are crucial to prevent future SA. Adolescence is a critical period for developing emotional regulation^42^, and adolescents’ NSSI and SA are associated with DER^43,44^. Hence, DER has a crucial impact on adolescents on the suicide continuum. NSSI is more common in early adolescence, whereas SA are more prevalent in late adolescence, leading to suicide in mid-adulthood^45^. Therefore, proactive intervention to reduce DER during adolescence can help lower future suicide risk. Cognitive behavioral therapy (CBT) and dialectical behavior therapy (DBT) for adolescents are the most effective methods for treating adolescent NSSI and SA by intervening in DER^46,47^. Implementing suicide prevention measures in middle and high schools can effectively reduce SA among adolescents^48^.

Our study has several limitations. First, it was a cross-sectional study that utilized a non-clinical sample and relied on self-report measures. Additionally, the data were collected during the COVID-19 period, which might have influenced the participants’ emotional experiences. Moreover, while we collected data on mobile phone addiction and empathy in the survey, stepwise regression analysis did not show a strong correlation with NSSI and SA. Furthermore, the threshold effect of DER on the risk of SA in adolescents with NSSI was not significant. Despite these limitations, our study employed a large sample size to explore the psychological mechanisms and significance of DER in influencing NSSI and SA among Chinese adolescents. We identified the intervention and prevention value of DER, thereby contributing to the theoretical research in the field of adolescent suicide. Moving forward, we plan to conduct longitudinal studies to further investigate the clinical significance of DER among Chinese adolescents exhibiting NSSI and SA behaviors. Interventions for DER in adolescents will also be a major focus of our future research. Given the significant role DER in adolescent NSSI and SA, future research should focus on the development and evaluation of interventions specifically targeting DER, particularly for adolescents facing emotional regulation challenges. As this study primarily focused on the adolescent population in China, future studies could expand to cross-cultural investigations to examine how DER influences adolescent NSSI and SA across different cultural contexts. It is also recommended that early screening of emotional regulation skills be conducted within schools and communities, alongside the provision of emotional regulation training or psychological support for high-risk adolescents to reduce the incidence of NSSI and suicidal behaviors. Beyond traditional CBT and DBT, innovative, personalized interventions, such as virtual reality-based therapy and AI-assisted emotional regulation training, should be further explored. Future research should encourage greater interdisciplinary collaboration between psychology, sociology, and clinical medicine to offer a comprehensive understanding of the relationship between DER and adolescent NSSI and SA, thereby providing a more robust theoretical foundation for intervention design.

## Electronic supplementary material

Below is the link to the electronic supplementary material.

Supplementary Material 1

## Data Availability

The data that support the findings of this study are available from the corresponding author upon reasonable request.
