# Supplementary material for: The role of difficulties in emotion regulation on non-suicidal self-injury and suicide attempts: a cross-sectional study of Chinese adolescents
Source: Sci Rep. 2025 Jul 1;15:21620. doi: 10.1038/s41598-025-91962-5 (PMC12216061; doi:10.1038/s41598-025-91962-5)
Supplement: Supplementary file 1 — Supplementary Material 1 [file 41598_2025_91962_MOESM1_ESM.docx]

# Supplementary Material

## Table S1. Harman’s single factor test

| Component | **Initial eigenvalues** | | | **Extraction sums of squared loadings** | | |
| --- | --- | --- | --- | --- | --- | --- |
|  | Total | % of Variance | Cumulative % | Total | % of Variance | Cumulative % |
| 1 | 2.892 | 36.148 | 36.148 | 2.892 | 36.148 | 36.148 |
| 2 | 1.045 | 13.060 | 49.207 | 1.045 | 13.060 | 49.207 |
| 3 | 1.027 | 12.837 | 62.044 | 1.027 | 12.837 | 62.044 |
| 4 | .898 | 11.227 | 73.271 |  |  |  |
| 5 | .691 | 8.632 | 81.904 |  |  |  |
| 6 | .557 | 6.960 | 88.864 |  |  |  |
| 7 | .496 | 6.200 | 95.063 |  |  |  |
| 8 | .395 | 4.937 | 100.000 |  |  |  |

Extraction Method: Principal Component Analysis.

## Table S2. Correlation analysis

|  | **Correlation coefficient（r）** | | |  |  |  |
| --- | --- | --- | --- | --- | --- | --- |
| **Variables** | DERS | PHQ-9 | IRI | MPATS | NSSI | SA |
| DERS | 1 | - | - | - |  | - |
| PHQ-9 | 0.601^***^ | 1 | - | - |  | - |
| IRI | 0.320^***^ | 0.315^***^ | 1 | - |  | - |
| MPATS | 0.415^***^ | 0.360^***^ | 0.297^***^ | 1 |  | - |
| NSSI | 0.448^***^ | 0.450^***^ | 0.174^***^ | 0.245^***^ | 1 | - |
| SA | 0.264^***^ | 0.276^***^ | 0.153^***^ | 0.161^***^ | 0.310^***^ | 1 |

r: Spearman's correlation coefficient； ^***^*p* < 0.001.

## Table S3. Stepped-linear regression model of NSSI

|  |  | |  |  |  |  |  | **Collinearity Diagnostics** | |
| --- | --- | --- | --- | --- | --- | --- | --- | --- | --- |
| **Models** | **Variables** | ***β***（Unstandardized） | | ***β***（Standardized） | ***t*** | ***p*** | **95% CI** | **Tol** | **VIF** |
| 1 | Constant | | -0.068 |  | -3.184 | 0.001 | (-0.109, -0.026) |  |  |
|  | PHQ-9 | | 0.565 | 0.478 | 24.546 | <0.001 | (0.520, 0.610) | 1.000 | 1.000 |
| 2 | Constant | | -0.068 |  | -3.286 | 0.001 | (-0.109, -0.027) |  |  |
|  | PHQ-9 | | 0.418 | 0.354 | 15.576 | <0.001 | (0.366, 0.471) | 0.700 | 1.429 |
|  | DERS | | 0.244 | 0.024 | 9.983 | <0.001 | (0.196, 0.292) | 0.700 | 1.429 |

Stepwise-forward;

## Table S4. Stepwise logistic regression model of suicide attempts

| **Models** | **Variables** | ***β*** | ***S.E.*** | ***p*** | ***OR*** | **95% CI** |
| --- | --- | --- | --- | --- | --- | --- |
| 1 | NSSI | 0.088 | 0.006 | <0.001 | 1.092 | (1.079, 1.106) |
|  | constant | -1.872 | 0.071 | <0.001 | 0.154 |  |
| 2 | NSSI | 0.064 | 0.007 | <0.001 | 1.066 | (1.052, 1.080) |
|  | PHQ-9 | 0.084 | 0.010 | <0.001 | 1.087 | (1.066, 1.109) |
|  | constant | -2.559 | 0.114 | <0.001 | 0.077 |  |
| 3 | NSSI | 0.064 | 0.007 | <0.001 | 1.066 | (1.052, 1.081) |
|  | PHQ-9 | 0.077 | 0.010 | <0.001 | 1.080 | (1.059, 1.102) |
|  | Sex (1) | 0.623 | 0.108 | <0.001 | 1.864 | (1.507, 2.305) |
|  | constant | -2.867 | 0.131 | <0.001 | 0.057 |  |
| 4 | NSSI | 0.058 | 0.007 | <0.001 | 1.059 | (1.045, 1.074) |
|  | PHQ-9 | 0.057 | 0.012 | <0.001 | 1.059 | (1.035, 1.083) |
|  | DERS | 0.013 | 0.004 | <0.001 | 1.013 | (1.006, 1.020) |
|  | Sex (1) | 0.600 | 0.109 | <0.001 | 1.822 | (1.472, 2.254) |
|  | constant | -3.879 | 0.319 | <0.001 | 0.021 |  |

Stepwise: conditional.

## Table S5. ≥87 and < 87 points of emotional regulation difficulty, 12 kinds of NSSI behavior

|  | **<87, *N*=782** | | **≥87, *N* = 1,714** | |  |
| --- | --- | --- | --- | --- | --- |
| **Variables** | ***N*** | **Total** | ***N*** | **Total** | ***Z*** |
| **Self-pinching** | 266 | 358 | 1,037 | 1,781 | 13.534^***^ |
| **Self-scratching** | 187 | 251 | 925 | 1,619 | 14.887^***^ |
| **Headbutting hard objects** | 141 | 185 | 684 | 1,151 | 11.405^***^ |
| **Punching hard surfaces** | 512 | 702 | 1,201 | 2,063 | 6.161^***^ |
| **Self-striking** | 140 | 176 | 711 | 1,198 | 12.176^***^ |
| **Self-biting** | 146 | 186 | 668 | 1,136 | 10.737^***^ |
| **Hair-pulling** | 112 | 149 | 516 | 873 | 8.868^***^ |
| **Self-piercing/stabbing** | 68 | 90 | 490 | 860 | 11.337^***^ |
| **Self-cutting** | 125 | 167 | 654 | 1,117 | 11.556^***^ |
| **Self-burning/scalding** | 21 | 27 | 242 | 424 | 8.700^***^ |
| **Skin rubbing to bleeding** | 66 | 80 | 445 | 789 | 10.428^***^ |
| **Skin carving(tattoos excluded)** | 100 | 128 | 521 | 867 | 9.853^***^ |

^***^*p* < 0.001

## Table S6. Threshold effects analysis on risk of SA

| **Models** | ***OR*** | **95% CI** | ***p*** |
| --- | --- | --- | --- |
| **Model 1** |  |  |  |
| Single OR | 1.198 | (1.048, 1.368) | 0.008 |
| **Model 2** |  |  |  |
| Turning point (K) 121 | 121 |  |  |
| <K (a) | 1.289 | (1.101, 1.509) | 0.002 |
| ≥K (b) | 0.806 | (0.506, 1.282) | 0.362 |
| Log-likelihood ratio test |  |  | 0.082 |

##
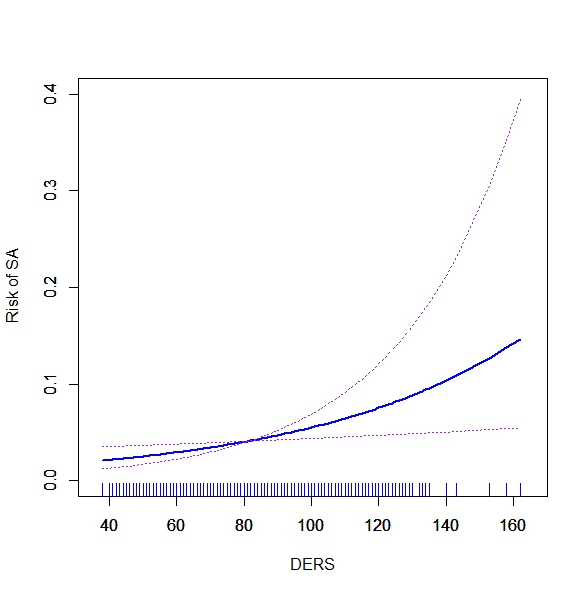


## Figure S1. The smoothing splines of DER and SA in adolescents with NSSI
